# Immunohistochemical analyses on two distinct internodes of stinging nettle show different distribution of polysaccharides and proteins in the cell walls of bast fibers

**DOI:** 10.1007/s00709-021-01641-1

**Published:** 2021-04-10

**Authors:** Claudia Faleri, Xuan Xu, Lavinia Mareri, Jean-Francois Hausman, Giampiero Cai, Gea Guerriero

**Affiliations:** 1 Dipartimento Scienze della Vita, University of Siena, via Mattioli 4, Siena, Italy; 2 Environmental Research and Innovation (ERIN) Department, Luxembourg Institute of Science and Technology (LIST), Hautcharage, Luxembourg

**Keywords:** Nettle, Secondary cell wall, Bast fibers, Fiber crops, Polysaccharide epitopes

## Abstract

Stinging nettle is a perennial herbaceous species holding value as a multi-purpose plant. Indeed, its leaves and roots are phytofactories providing functional ingredients of medicinal interest and its stems produce silky and resistant extraxylary fibers (a.k.a. bast fibers) valued in the biocomposite sector. Similarly to what is reported in other fiber crops, the stem of nettle contains both lignified and hypolignified fibers in the core and cortex, respectively, and it is therefore a useful model for cell wall research. Indeed, data on nettle stem tissues can be compared to those obtained in other models, such as hemp and flax, to support hypotheses on the differentiation and development of bast fibers. The suitability of the nettle stem as model for cell wall-related research was already validated using a transcriptomics and biochemical approach focused on internodes at different developmental stages sampled at the top, middle, and bottom of the stem. We here sought to complement and enrich these data by providing immunohistochemical and ultrastructural details on young and older stem internodes. Antibodies recognizing non-cellulosic polysaccharides (galactans, arabinans, rhamnogalacturonans) and arabinogalactan proteins were here investigated with the goal of understanding whether their distribution changes in the stem tissues in relation to the bast fiber and vascular tissue development. The results obtained indicate that the occurrence and distribution of cell wall polysaccharides and proteins differ between young and older internodes and that these changes are particularly evident in the bast fibers.

**Supplementary Information:**

The online version contains supplementary material available at 10.1007/s00709-021-01641-1.

## Introduction

Currently, the exploitation of plant biomass as a source of energy and of added-value molecules for industrial applications is a pressing demand to promote a bioeconomy that is both sustainable and environmentally friendly. Herbaceous crops are important renewable resources because they produce biomass much faster than woody species, given the shorter time required to complete their life cycle (Debnath 2015; Andre et al. 2016). Among herbaceous crops, plants that produce bast fibers are particularly interesting because they supply both long and strong fibers containing considerable amounts of crystalline cellulose. Examples of such plants are flax and hemp. Bast fibers are used not only in the textile industry but also by the biocomposite sector as eco-friendly alternatives to artificial fibers. While species such as flax and hemp have gained remarkable interest from the scientific community, nettle (*Urtica dioica* L.) has not been studied as much, hence it is considered one of the most underrated plants among those of potential economic interest (Di Virgilio et al. 2015). Proposing nettle as a model system also arises from the ease of cultivation, its low environmental impact, and low cost of management. In addition, the availability of its transcriptome and of high-throughput RNA-Seq datasets (Xu et al. 2019; Carpenter et al. 2019) favors molecular comparisons with other fiber crop models.

Nettle subsp. *dioica* is a perennial dioecious plant that grows in temperate regions and is a rich source of phytochemicals (Grauso et al. 2020). Additionally, it is resistant to several biotic stressors and is considered a weed since its fast development is guaranteed by a well-developed system of roots and rhizomes. The cultivation of multi-purpose plants has applications in various sectors including textiles, biocomposites, and therapeutics; indeed, the secondary metabolites produced by these plants have different hemostatic, anti-inflammatory, and diuretic properties (Hudec et al. 2007; Johnson et al. 2013). The nettle stem contains a woody core surrounded by a cortex with long bast fibers (Bacci et al. 2009) that are highly resistant to mechanical stress (Bodros and Baley 2008). In addition, the stem has a gradient of lignification from the top to the bottom; this progressive lignification is necessary for the mechanical strength of the basalmost internodes of the stems. The process of progressive girth increase accompanies, basipetally, the sequential stages of fiber development that include elongation and thickening of the cell wall (Backes et al. 2018).

In hemp, flax, and nettle, bast fibers are usually characterized by thick gelatinous cell walls (called G-layers) (Chernova et al. 2018) which are made up of crystalline cellulose. This type of cell walls is different from the xylan-type cell walls found in jute and kenaf fibers (Mikshina et al. 2013; Guerriero et al. 2017). The mechanical properties of the bast fibers are determined by the order of assembly of macromolecules (polysaccharides, lignin, and proteins) in the different layers of the cell walls, according to a precise temporal development that involves the synthesis of the primary cell wall (PCW), then the development of the secondary cell wall (SCW) and, ultimately, of the G-layer. The composition of the S1 layer of the SCW is like the xylan-type cell wall, while the G-layer contains a high cellulose content (whose fibrils are axially oriented and contain neither xylan nor lignin) and is characterized by a remarkable thickness (Mikshina et al. 2013). The G-layer has similarities to the cell wall of tension wood that has lower amounts of hemicellulose and lignin (Mellerowicz and Gorshkova 2012). Since the G-layer confers the most desired mechanical properties, an in-depth analysis of its composition and developmental plan is of paramount importance.

The progressive top-to-bottom increase in stiffness and thickness (linked to the thickening of bast fibers and the progressive stem lignification) makes nettle an ideal model to study the events regulating secondary growth and bast fiber formation. Understanding these mechanisms at the molecular and cytological/ultrastructural level may help identify those aspects that are most critical and suitable to improve the tensile properties of bast fibers.

Previously, a transcriptomic analysis of nettle “clone 13” (a fiber-clone) was performed to improve nettle knowledge and promote its exploitation on a longer-term perspective. The transcriptomic analysis was conducted both on whole internodes sampled from the top and middle of the stem and on cortical and core tissues in the bottom internode (Xu et al. 2019). The aim was to compare the different gene expression patterns accompanying the development of bast fibers and the increased basipetal lignification. As expected, the bottom internode was enriched in the expression of genes taking part in distinct aspects of SCW synthesis (such as cellulose, hemicellulose, lignin biosynthesis, as well as aromatic amino acids’ production and sucrose transporters). In contrast, the top and middle internodes showed a higher expression of genes related to phytohormone metabolism and cell expansion.

Chemical analysis of the nettle stem showed a difference in composition when comparing whole internodes (hence comprising both cortical peels and central core tissues) from the top and middle regions of the stem with the peels of the bottom part. Older internodes (containing bast fibers with a thicker G-layer) exhibited higher levels of galactose and increased presence of Ca^2+^-pectate gels than those from the top and middle regions. Moreover, preliminary ultrastructural analyses revealed the presence of a G-layer in the cell wall of bast fibers showing a layered structure. This layer shows cross-reaction with a peptide recognizing crystalline cellulose and has a loose appearance, with regions where the layer seems to be flaking off (Xu et al. 2019).

Understanding the processes related to the progressive strengthening of the nettle stem also requires a visual analysis of the ultrastructural events in two distinct zones of stem development (a more flexible internode *vs* a thicker and stiffer internode); the latter is characterized by a larger number of bast fibers and a differential accumulation of cell wall polysaccharides. As already done in flax and hemp, we believed it necessary to provide a detailed analysis of the composition/distribution of polysaccharides in bast fibers of two distinct nettle internodes. Analyzing the distribution of specific cell wall polysaccharides can help understand the developmental pattern of bast fibers in terms of polysaccharide composition in the two distinct stem internodes; those regions correspond to zones of primary (that is, younger, flexible internodes) and secondary growth (that is, older, more lignified ones). The results will complement the molecular data previously obtained (Xu et al. 2019) and may allow comparison with information obtained in other fiber crops. For this purpose, we have used specific antibodies, extensively described in the literature, and directed against specific epitopes recognizing cell wall polysaccharide backbones and side chains. The immunohistochemical analyses were performed on the top and bottom internodes to focus on two stem tissues with contrasting cell wall chemical and mechanical properties.

## Materials and methods

### Sampling of nettle stem in two distinct areas

**Fig. 1:**
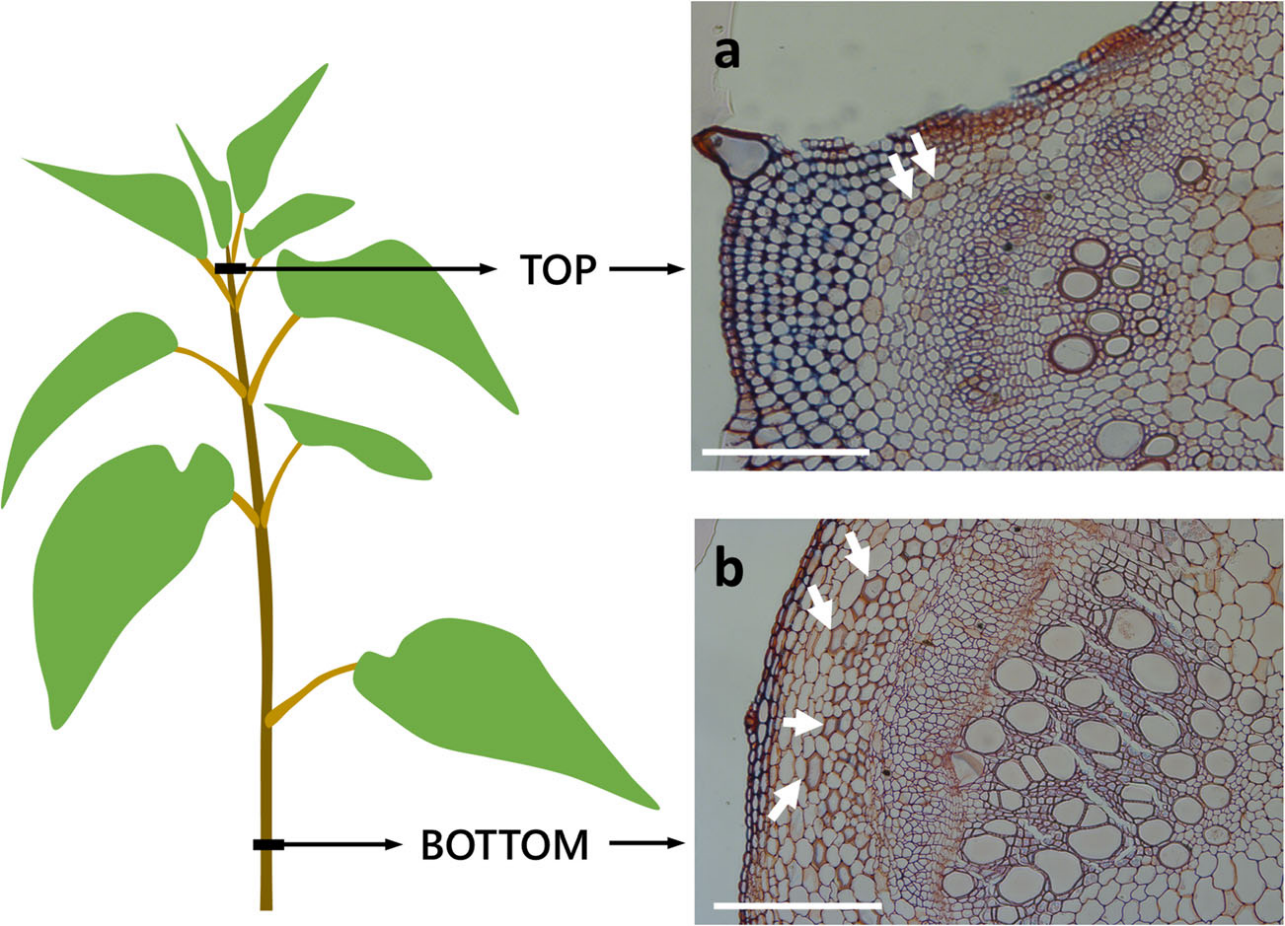
Schematic representation of a nettle plant with the two regions analyzed in this work: the “top” internode, more flexible and elastic, and the “bottom” internode, thicker and stiffer. Histological images on the right show sections of the nettle stem at the top (**a**) and bottom level (**b**); at the top level, developing bast fibers are indicated by arrows; in the case of bottom stem, bast fibers with thick cell wall are also indicated by arrows. The images are fixed sections stained with FASGA. Bars: 200 μm

Nettle plants were propagated through stem cuttings and were grown in incubators with a cycle of 16 h of light at 25 °C and 8 h of darkness at 20 °C, as previously described (Xu et al. 2019). After 5 weeks, the stem internodes were collected from two regions positioned right below the internode containing the apical meristem (top) and two internodes below the snap point (bottom) (Fig. 1). Two biological replicates, corresponding to two independent plants, were sampled and analyzed. At the level of top internode, bast fibers have been identified following a positional-based approach; essentially, we first focused on vascular bundles, hence we moved on the region immediately outside the phloem (just below the cortex) where two cell types could be essentially found, one of parenchymatous type with only PCW and a cell type showing onset of SCW formation. The latter were identified as bast fibers.

### Antibodies

The following antibodies from Plant-Probes (http://www.plantprobes.net) were used: LM5 (specific for (1-4)-β-D-galactan), LM16 (galactosyl residues of (1-5)-α-L-arabinan backbone), JIM5 (partially methyl-esterified/un-esterified homogalacturonan), JIM7 (methyl-esterified homogalacturonan), LM14 (arabinogalactan proteins), and LM20 (methyl-esterified homogalacturonan). The catalogue number of antibodies corresponds to the name of the antibodies themselves. The antibodies INRA-RU1 and INRA-RU2 (for the backbone of rhamnogalacturonan I, with RU1 requiring at least 6 rhamnose-galacturonic acid repeats and RU2 a minimum of two) were kindly provided by Dr. Marie-Christine Ralet (INRA Angers-Nantes). The selection of antibodies was motivated by the biochemical and transcriptomics data already generated (Xu et al. 2019).

### Confocal microscopy

The sample preparation for the immunohistochemical analysis with confocal microscopy was performed as previously described (Backes et al. 2018). Briefly, 5-mm-thick sections collected from top and bottom internodes were vacuum-impregnated in the fixative solution containing 1% (w/v) glutaraldehyde, 2% (w/v) paraformaldehyde, 1% (w/v) caffeine, and 0.2 M sodium phosphate buffer (pH 7.2). After fixing overnight at 4 °C, the samples were dehydrated in an ethanol series (v/v): 70% for 30 min, 70% for 60 min, 95% for 30 min, 95% for 60 min, and 100% for 30 min. The samples were then embedded in the resin containing 2% (v/v) PEG 400 and 0.4% (w/v) dimethacrylate ethylene glycol and then included. Cross sections of 10 μm thickness were obtained using a microtome (Leica) and placed on glass slides. The sections were incubated with diluted antibodies (10-fold dilution for LM14/20 and 5-fold dilution for INRA-RU1/2) for 1.5 h, washed three times with PBS, and then incubated for 1.5 h with 100-fold diluted secondary antibodies (Sigma; anti-rat IgG coupled to FITC for LM14/20 and anti-mouse IgG coupled to FITC for INRA-RU1/2). The sections were rinsed three times with PBS, mounted in 50% (v/v) glycerol, and analyzed by a Zeiss LSM 880 confocal microscopy (Carl Zeiss AG). The wavelength of excitation and emission was set at 488 and 602 nm, respectively.

### Sample preparation for electron microscopy

All samples were fixed with 3% (v/v) glutaraldehyde in cacodylate buffer for 2 h at room temperature and then left at 4 °C overnight. After washing in cacodylate buffer (3 times for 15 min each), specimens were post-fixed with 1% (w/v) OsO_4_ for 1 h at room temperature. After at least 3 washes in water (15 min each), specimens were stored in water until dehydration in a graduated ethanol series: 10% (v/v) EtOH for 5 min, 30% EtOH for 10 min, 50% EtOH for 15 min, and 70% EtOH for storage. After dehydration, sections were included in the Spurr’s resin for ultrastructural analysis. Ultrafine sections were obtained with a diamond knife in the ultra-microtome LKB NOVA, collected on a grid, and stained for 20 min with 2% (w/v) uranyl acetate and for 5 min with lead citrate at room temperature (Reynolds 1963).

### Sample preparation for immunoelectron microscopy

The nettle stem was dissected at the top and bottom regions with a sharp razor blade. Sections (no thicker than 2 mm) were fixed for 2 h at room temperature and left overnight at 4 °C in a mixture of 2% (v/v) glutaraldehyde and 1.6% (v/v) paraformaldehyde in 0.1 M phosphate buffer pH 6.9. Subsequently, specimens were washed with phosphate buffer (2 × 10 min) and then dehydrated in a series of absolute ethanol at the following percentages (v/v): 10% for 5 min, 30% for 10 min, 50% for 15 min, 70% for 30 min, and 100% for 1 h (with changes every 20 min). Specimens were infiltrated with LR-White resin in the following ratio with ethanol: 1:1 resin:ethanol overnight, 3:1 resin:ethanol for 1 day, and pure resin for 1 day. The resin was polymerized for 2 days at 40 °C. Ultrafine sections were obtained with a diamond knife using the ultra-microtome LKB NOVA.

### Immunolocalization

For immunolocalization, the nettle stem sections were collected on gold grids and blocked for 20 min with normal goat serum (NGS) diluted 1:30 in dilution buffer (0.05 M Tris-HCl pH 7.6, 0.9% w/v NaCl, and 0. 2% w/v BSA). Sections were incubated for 4 h at room temperature with primary antibodies. Antibodies were diluted 1:5 with dilution buffer. Sections were then washed for 20 min in the dilution buffer with 0.1% (v/v) Tween 20 and incubated for 45 min at room temperature with either secondary anti-rat antibodies (for LM5, LM16, JIM5, JIM7, LM14, and LM20) or anti-mouse antibodies (for RU1 and RU2) conjugated to 10 nm gold particles and diluted 1:20 in 0.02 M Tris-HCl pH 8.2. All sections were examined with the Philips MORGAGNI 268 80-kV transmission electron microscope, equipped with MEGAview II camera, and were processed with the Analysis software. Controls were carried out by omitting the primary antibodies; in any case, we have never detected signals due to secondary antibodies. Examples of control images are present as Supplementary Fig. S1.

### Image analysis

To quantify the distribution of gold particles (and therefore the antibody signal), immunolocalization images were measured by signal density. Images were imported into ImageJ (https://imagej.nih.gov/ij) and their size was calibrated with the “Set Scale” command. Subsequently, images were subjected to “Threshold” to highlight only the gold particles. Using the “Straight Line” command, a “line of interest” was drawn in the regions of the cell wall where the density of gold particles should be measured. The thickness of the line of interest was extremely wide (about 500 nm) to obtain more representative data. We usually analyzed 10 images per sample by making 3–4 measurements for each of them. The data were imported together (“Add to Manager” option) and then compared using the MultiPlot command. The ordinate axis was indicative of the relative density of the gold particles, then of the antibody signal, while the abscissa axis reported the distance from the plasma membrane of bast fiber cells. The data were then exported as comma separated values, imported into Microsoft Excel, and then rendered as chart.

### Statistical analysis

All experiments (both immunofluorescence and immunoelectron microscopy analyses) were repeated independently three times with equivalent results.

## Results

### Ultrastructural analysis of the cell wall of bast fiber cells

**Fig. 2:**
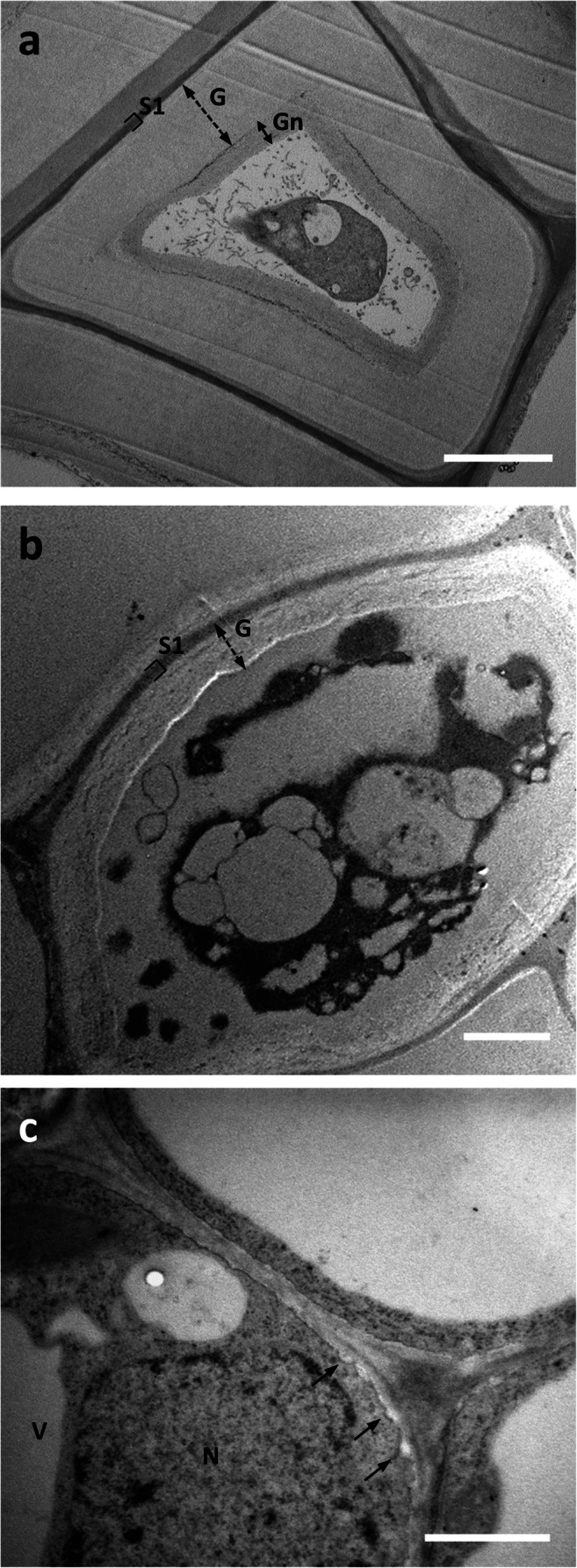
Comparative analysis by transmission electron microscopy of bast fibers in the hemp hypocotyl (after 20 days) and nettle stem. **a** Ultrastructure of a bast fiber in the stem of hemp. Note the thick G-layer (double dashed arrow) and the inner Gn-layer (double arrow). Bar: 2 μm. **b** A bast fiber in the nettle stem sampled at the bottom internode; apart from the different relative thickness, the G-layer appears as multi-layered and visually less compact. Bar: 2 μm. Both cells are characterized by cytoplasmic residues in the center. **c** A developing bast fiber at the level of the top internode. A thinner cell wall can be observed but characterized by the presence of local thickenings. N, nucleus. V, vacuole. Bar: 1 μm

Ultrastructural observations of the cell wall of bast fibers sampled at the bottom of the stem confirmed, as previously shown (Xu et al. 2019), a distinctive structure. Unlike other comparable fibers (such as those of flax or hemp), the G-layer appeared much less compact, as if it consisted of several layers stuck to each other (Fig. 2, compare image 2a with 2b, the G-layer is indicated by the double dashed arrow). We decided to use the term “multi-layered” to define this type of cell wall. The presence of layers could be observed across the G-layer, without any specific distinction between regions closest to the plasma membrane and more distant region. The G-layer was laid as a series of successive layers whose compactness was difficult to assess because microscopy images showed cleavage or detachment of the various layers (see also TEM images in Xu et al., 2019). Although detachment of the various layers can be interpreted as an artifact of the microscopy procedure, the evidence that it did not occur in other species, such as hemp (Behr et al. 2019), can be taken as an index of lower compactness. This type of G-layer looked quite different when compared to the more homogenous and compact type of flax and hemp bast fibers. It appeared also different from the stripped and fibrillar Gn-layer previously described in hemp (Chernova et al. 2018; Behr et al. 2019). By comparison, a TEM image of a developing bast fiber is shown (Fig. 2c); in this case, it is possible to observe a thinner cell wall characterized by the appearance of local thickenings (arrows) that can be interpreted as the beginning of the SCW deposition.

### Distribution of LM5-labeled galactans and LM14-labeled arabinogalactan proteins

**Fig. 3:**
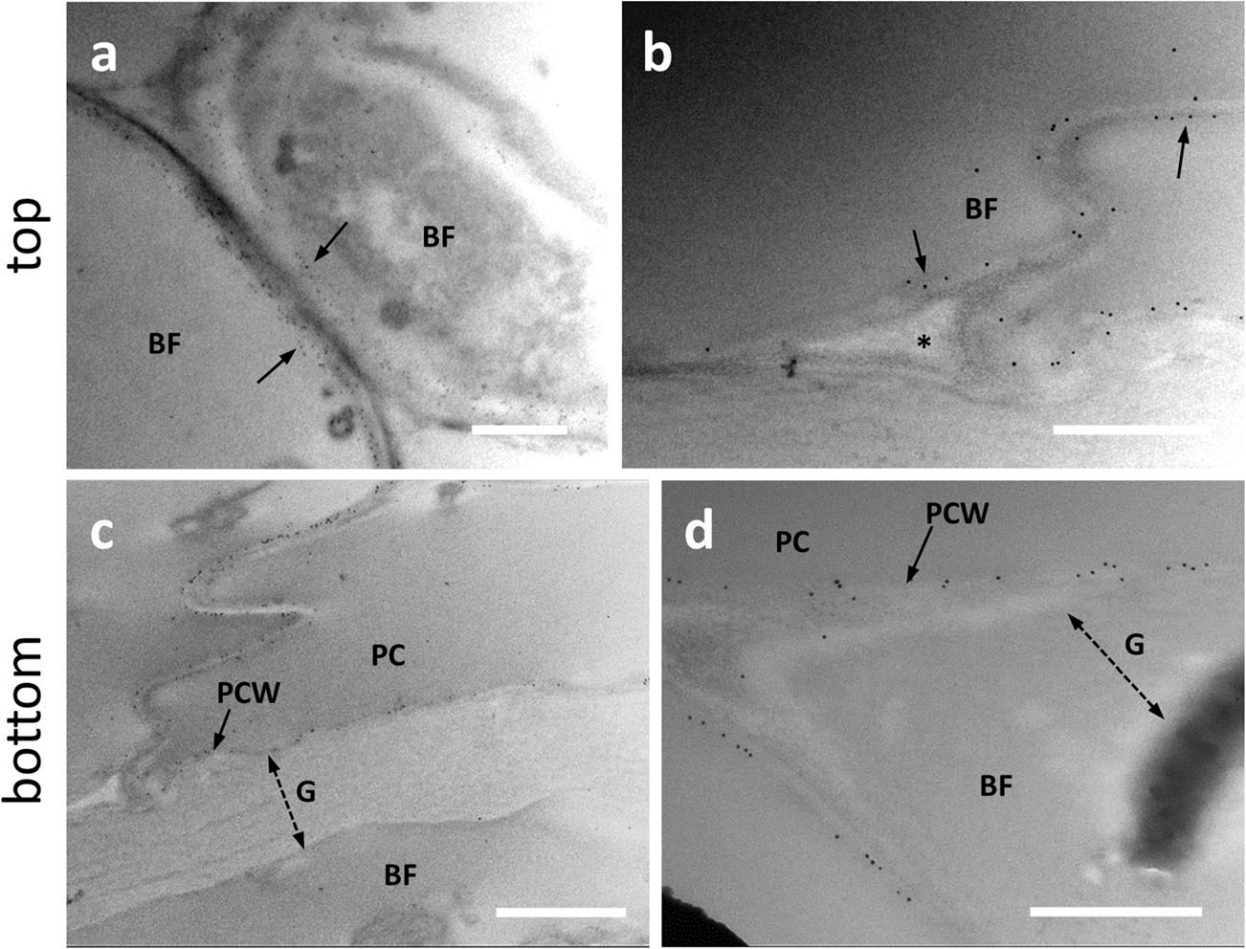
Distribution of the epitope recognized by the antibody LM5 directed against galactans in the top (**a**–**b**) and bottom internodes (**c**–**d**) of nettle stem. **a** Bast fibers (BF) at the beginning of the differentiation process. The LM5 antibody signal can be detected between the cytoplasm and the cell wall (arrows). Bar: 1000 nm. **b** A detail of bast fibers (BF) being formed. Arrows indicate the location of the antigen at the boundary between cytoplasm and cell wall. The signal is absent in intercellular spaces (asterisk). Bar: 500 nm. **c** A bast fiber (BF) at the bottom internode next to a parenchyma cell (PC) whose primary cell wall (PCW) is labeled. The thick G-layer of bast fibers, indicated by the double dashed arrow, is devoid of LM5 signal. Bar: 1000 nm. **d** Another example of bast fiber (BF) with no signal in the thick G-layer (double dashed arrow). The signal is intense in the primary cell wall (PCW, arrow) of the adjacent parenchyma cell (PC). Bar: 500 nm

Galactans are galactose-containing polymers that are synthesized simultaneously with cellulose; in flax, post-synthetic modifications of galactans increase the crystallinity of cellulose thereby promoting the transition from Gn-layer to G-layer (Gorshkova and Morvan 2006; Roach et al. 2011). Here, we investigated the distribution of these polymers using the antibody LM5. The signal intensity observed in the top sections was generally weak (Fig. 3). However, the signal was very often associated with the (primary) cell wall of developing bast fibers (Fig. 3a, arrows). The signal appeared to be associated with the cytoplasmic side of the cell wall; as suggested by Fig. 3b (arrows), we cannot even exclude an association with the plasma membrane. The LM5 antibody signal was never found in the intercellular spaces (indicated by the asterisk in Fig. 3b). In the bottom sections of nettle stems (Fig. 3), the LM5 signal in the G-layer of bast fibers (BF) was difficult to observe or even absent. For comparison, Fig. 3c clearly shows LM5 signal in the primary cell wall (PCW) of parenchyma cells (PC) and the absence of signal in the G-layer of bast fibers (BF), which is again indicated by the double dashed arrow. The result in Fig. 3d confirms the presence of the LM5-recognized epitope in the PCW of parenchyma cells (PC) and the absence of LM5 signal in the thick G-layer of bast fibers. We could hypothesize an association of the LM5 signal also with the PCW of bast fibers, but the results obtained were not convincing. We conclude that the distribution of the epitope recognized by LM5 is chiefly associated with the PCW but not the G-layer of bast fibers.

**Fig. 4:**
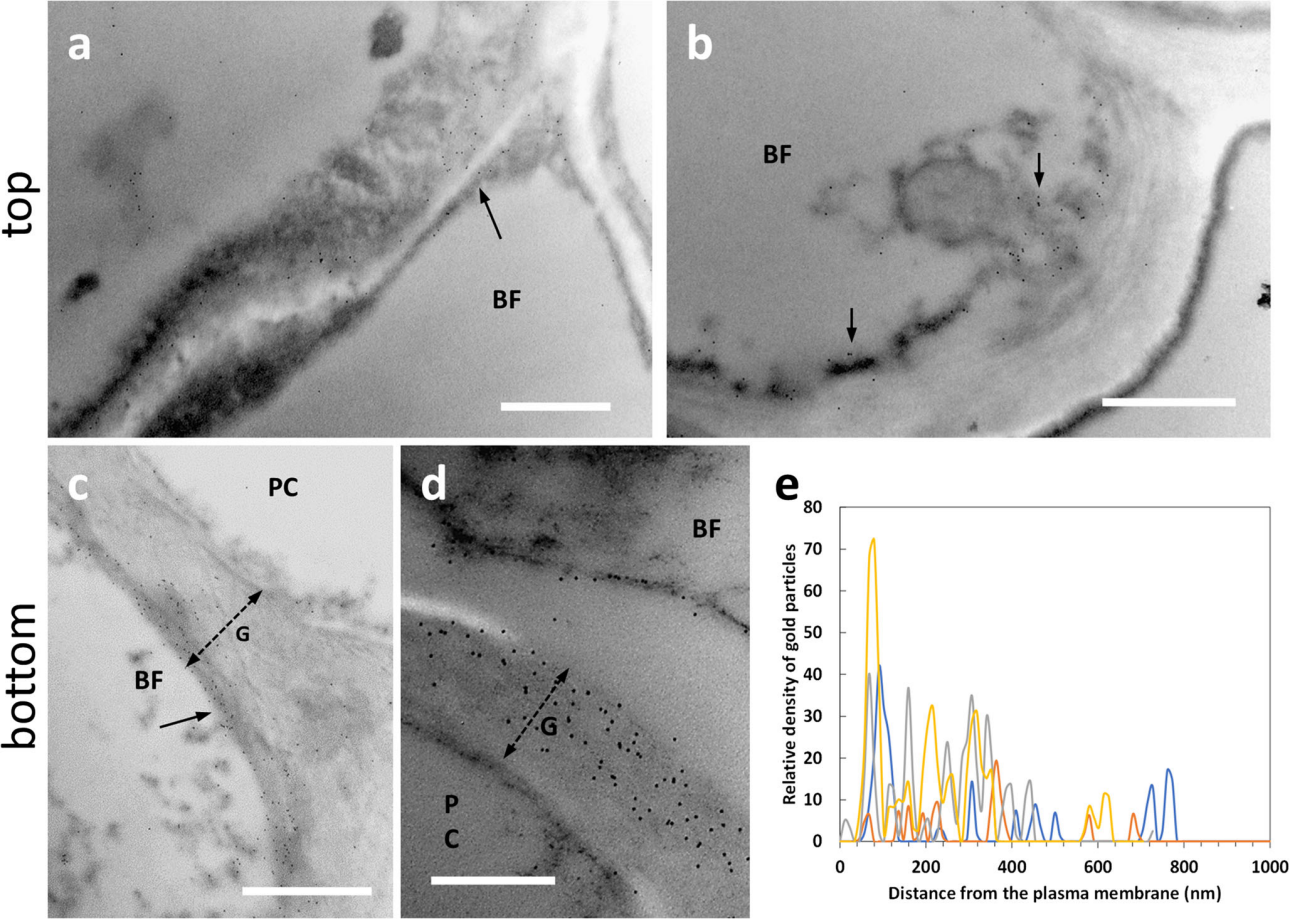
Distribution of the epitope recognized by the antibody LM14 directed against arabinogalactan proteins in the top (**a**–**b**) and bottom (**c**–**d**) internodes of nettle stem. **a** Detail of a developing bast fiber with an appreciable signal in the cell wall (arrow). Bar: 1000 nm. **b** A developing bast fiber (BF) with signal present both in the cell wall edge facing the cytoplasm and in membranous structures that cluster on the cell wall (arrows). Bar: 1000 nm. **c** A bast fiber (BF) sampled from a bottom internode with its thick G-layer (G, double dashed arrow). The LM14 signal is intense in the inner region of the cell wall facing the plasma membrane (arrow). PC indicates a parenchyma cell with little or no signal. Bar: 1000 nm. **d** A detail of a bast fiber (BF) from a bottom internode with its G-layer (G) strongly labeled in the half near the cell membrane as well as in the cytoplasm that is detached from the cell wall (arrow). PC, parenchyma cell. Bar: 500 nm. **e** Graph of LM14 signal distribution in bast fibers’ walls in relation to the distance from the plasma membrane. To make it easier to view the information, only four measurements were reported (in yellow, red, green, and gray)

Arabinogalactan proteins (AGPs) are associated with the plasma membrane, but the polysaccharide side chains protrude within the cell wall with which they interact. AGPs are glycoproteins involved in multiple aspects of cell development, including cell-to-cell communication and stress tolerance. We analyzed the presence of AGPs labeled by the antibody LM14. When we analyzed the top sections (Fig. 4), a weak LM14 signal was observed in the cell wall of developing bast fibers (BF) (arrow in Fig. 4a); by carefully observing the signal distribution in the cell wall, we assumed that the epitope of LM14 was mainly localized in the inner side of the G-layer (arrows in Fig. 4b), as if AGPs were progressively accumulated following the deposition of the cell wall. In the bottom sections (Fig. 4), labeling was observed in bast fibers, especially in the G-layer region facing the plasma membrane (Fig. 4c). This type of deposition is also confirmed by confocal microscopy (Supplementary Fig. S2). When bast fibers possessing thicker G-layers were analyzed (Fig. 4d), the LM14 signal could be found more widespread across the whole cell wall, but the inner side of the G-layer was always more intensively labeled (Fig. 4d). The signal was also found in association with the cytoplasm that in the image was detached (Fig. 4d, arrow). Densitometric analysis of the signal with ImageJ revealed that the epitope recognized by LM14 was present mainly in the region of the G-layer in contact with the plasma membrane (Fig. 4e), but then progressively decreased resulting very weak, if not completely absent. The comparison of data obtained in the top and bottom regions suggests that, as development advances, the LM14 signal increases in bast fibers due to the progressive deposition on the inner side of the cell wall G-layer.

### Distribution of LM16-labeled arabinans

**Fig. 5:**
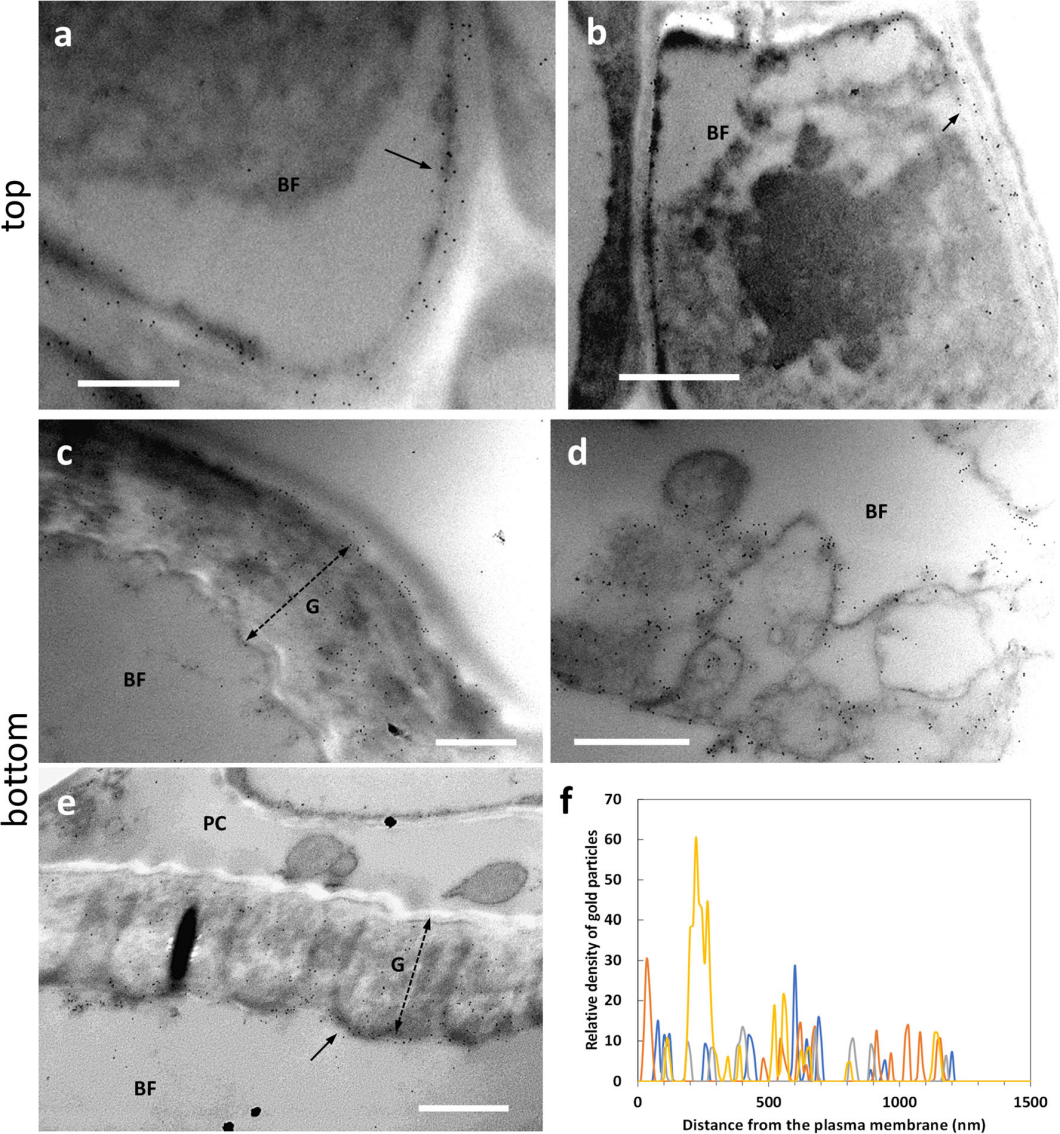
Distribution of the epitope recognized by the LM16 antibody directed against arabinans in the top (**a**–**b**) and bottom (**c**–**e**) internodes of nettle stem. **a** A developing bast fiber (BF) with evidence of LM16 signal in the inner half of the cell wall (arrow). Bar: 500 nm. **b** An additional image of a developing BF with the LM16 signal associated with the cell wall (arrow). Bar: 1000 nm. **c** A G-layer (double dashed arrow) of a bast fiber (BF) from a bottom internode intensely labeled by gold particles. The LM16 signal appears to be prevalent in the cytoplasm facing half of the cell wall. Bar 1000 nm. **d** Magnification of the cytoplasmic region of a bast fiber where a group of membranous structures is visibly labeled by the LM16 antibody. Bar: 1000 nm. **e** Another bast fiber (BF) with a thick G-layer (G, double dashed arrow) and a clear signal of LM16 in the half of the cell wall closest to the cytoplasm (arrow). Bar: 1000 nm. **f** Graph illustrating the distribution of LM16 signal in the secondary cell wall of bast fibers in relation to the distance from the plasma membrane

The distribution of arabinans was analyzed in both top and bottom sections using the antibody LM16. In top sections of nettle stem (Fig. 5), the LM16 signal was much less intense than the bottom samples. A certain amount of signal could be distinguished in the innermost side of the cell wall of bast fibers (BF, arrow in Fig. 5a). Figure 5b shows a developing bast fiber with indication (arrow) of the presence of the LM16 signal on the cytoplasmic side of the cell wall. These observations suggest that the epitope recognized by LM16 is most likely deposited according to the G-layer assembly scheme and therefore gradually accumulates with it. At the bottom level (Fig. 5), the LM16 signal was distributed in the thick G-layer (double dashed arrow) of bast fibers (BF). The signal did not show any specific localization and was spread throughout the G-layer (Fig. 5c). In the cytoplasm that remained in differentiated cells, the LM16 signal was associated, sometimes as clusters, with the endomembrane residues (Fig. 5d). Even when the signal was less intense (Fig. 5e), it was still found in the inner side of the G-layer (arrow). Distribution analysis with ImageJ showed that the epitope recognized by the LM16 antibody was evenly distributed in the first half of the G-layer relative to the plasma membrane, with occasional peaks in intensity (Fig. 5f); in the second half of the G-layer up to the PCW boundary, the LM16 signal was weak.

### Distribution of RU1-, RU2-, JIM7-, and JIM5-labeled pectins

**Fig. 6:**
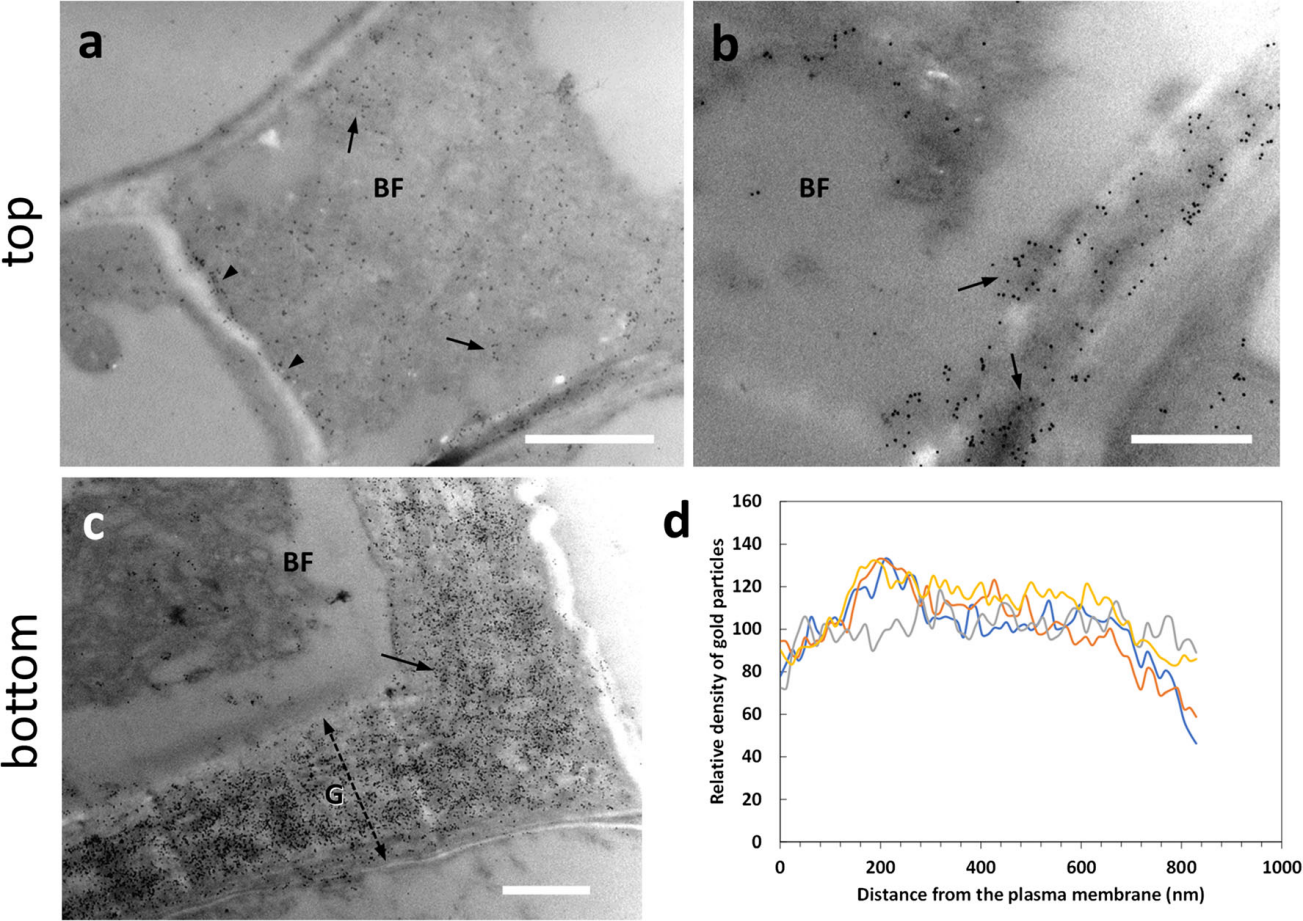
Distribution of the epitope cross-reacting with the antibody RU1 in the top (**a**–**b**) and bottom (**c**) internodes of nettle stem. **a** A bast fiber (BF) being developed. The RU1 antibody signal can be appreciated both in the cytoplasm (arrows) and at the cell wall (arrowheads). Bar: 1000 nm. **b** A detail of the boundary between cytoplasm and cell wall. The scattered distribution of the RU1 signal in the developing cell wall can be appreciated (arrows). Bar: 500 nm. **c** Detail of a bast fiber (BF) from a bottom internode where the remarkable presence of gold particles can be seen (arrow). The double dashed arrow indicates the thickness of the G-layer. Bar: 1000 nm. **d** Graph illustrating the distribution of the RU1 signal for the entire thickness of the bast fiber wall in relation to the distance from the plasma membrane

Due to the lack of an LM5 signal and to have more information on the distribution of pectic RG-I, an additional labeling was performed with the antibodies INRA-RU1 and INRA-RU2 (Ralet et al. 2010). When we analyzed the signal distribution of RU1 antibody in the top sections of nettle stem (Fig. 6), the signal showed a specific but still different distribution than in the bottom internode. Fig. 6a and b illustrates bast fibers at an early stage of development and both images show that the RU1 signal is both cytoplasmic (arrows) and associated with the cell wall (arrowheads). When examined in more detail, the RU1 signal appears to be diffusely present in the cell wall G-layer of bast fibers (Fig. 6b, arrows). Thus, the data of antibody labeling obtained in the top internode are indicative of a process of secretion from the cytoplasm and subsequent assembly into the cell wall. In the bottom region of nettle sections (Fig. 6), the RU1 signal was found to be highly abundant throughout the thickness of the bast fiber’s G-layer (Fig. 6c, arrow). This was confirmed by the strong specific labeling also observed by confocal microscopy (Supplementary Fig. S3a). The distribution of the RU1 signal appears very homogeneous, as also confirmed by densitometric analysis performed with ImageJ. As shown in Fig. 6d, the signal is remarkably constant across the entire G-layer, with a slight increase at some distance from the plasma membrane.

**Fig. 7:**
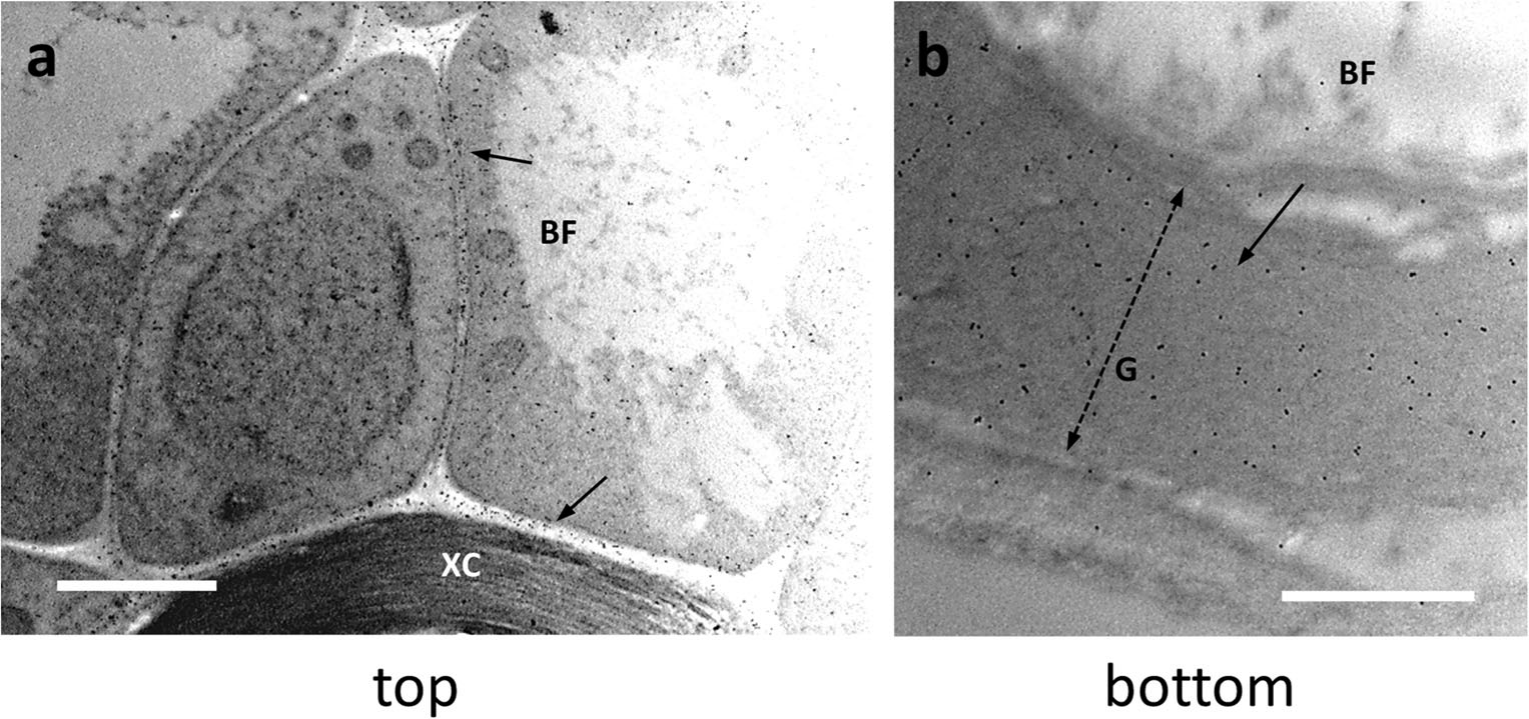
Distribution of the RU2 signal in the top (**a**) and bottom (**b**) internodes of nettle stem. **a** A developing bast fiber (BF), with clear signal in the cell wall (arrows). The underlying xylem cell (XC) does not show labeling with RU2. Bar: 2000 nm. **b** A bast fiber (BF) from a bottom internode with the typical multi-layered cell wall, clearly labeled by RU2 (arrow). The double dashed arrow indicates the thickness of the G-layer. Bar: 1000 nm

The distribution obtained with the RU2 antibody is quite comparable to that of RU1, both at the transmission electron (Fig. 7) and confocal microscope (Supplementary Fig. S3b). Although RU1 and RU2 cross-react with similar epitopes, it was considered appropriate to use the two antibodies both as control and in the light of the studies already described in hemp (Chernova et al. 2018; Behr et al. 2019). In developing bast fibers at the top internode, the RU2 signal was clear in the cell walls of BF (Fig. 7a, arrows) although a certain amount of signal was also detectable in the cell cytoplasm. By comparison, the xylem cell (XC) at the bottom of the image did not show any labeling. In nettle stem sections sampled at the bottom internode, the RU2 signal is quite intense in the G-layer of bast fibers (Fig. 7b, arrow). Although we did not make measurements of gold particle distribution, RU2 signal was dispersed in the G-layer.

**Fig. 8:**
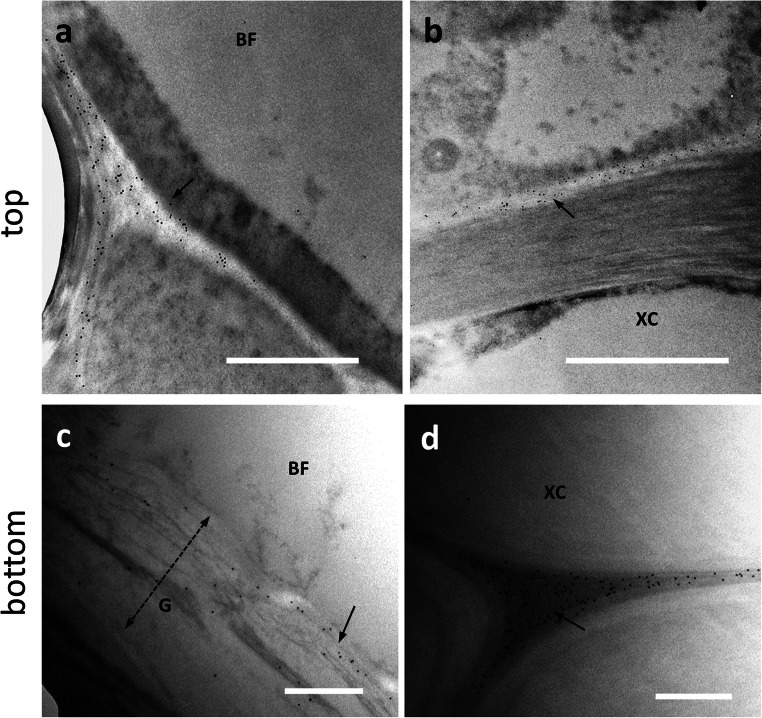
Distribution of the epitope recognized by the JIM5 antibody in the top (**a**–**b**) and bottom (**c**–**d**) internodes of nettle stem. **a** At the level of developing bast fibers, the JIM5 signal is weak and localized in the middle lamella or primary cell wall (arrow). Bar: 2000 nm. **b** By comparison, the cell wall of xylem cell (XC) does not show JIM5 signal, which is conversely present in the middle lamella and cell wall of adjacent cells. Bar 2000 nm. **c** In a bast fiber (BF) at the bottom internode, the JIM5 signal is appreciable but not intense and yet diffusely distributed (arrow). The double dashed arrow indicates the thickness of the G-layer. Bar: 500 nm. **d** For comparison, the JIM5 signal is found in the intercellular spaces between xylem cells (XC) (arrow). Bar: 500 nm

To get a broader picture of pectin distribution, we also analyzed the pectins recognized by the JIM5 antibody, i.e., acidic pectins (Fig. 8). Analysis of the JIM5 epitope in the top internode showed a labeling in PCWs and intercellular spaces. In the case of developing bast fibers, the signal was extremely weak and eventually confined in the middle lamella of adjacent cells (Fig. 8a, arrow) or in the PCWs. For comparison, we show the case of xylem cells (XC) where the JIM5 signal is again confined to the middle lamella or PCW (Fig. 8b, arrow). When we focused on bast fibers in the bottom internode (Fig. 8), the JIM5 signal was evident in the G-layer but not particularly intense (if compared with the signal of other antibodies); the distribution of the signal was rather diffuse and widespread in the cell wall (Fig. 8c, arrow). Even in this case, a comparison with xylem cells shows a significant difference: while in bast fibers, the epitope recognized by JIM5 is present in the G-layer, and in xylem cells (XC), the signal is found only in the intercellular spaces between adjacent cells (arrow in Fig. 8d).

**Fig. 9:**
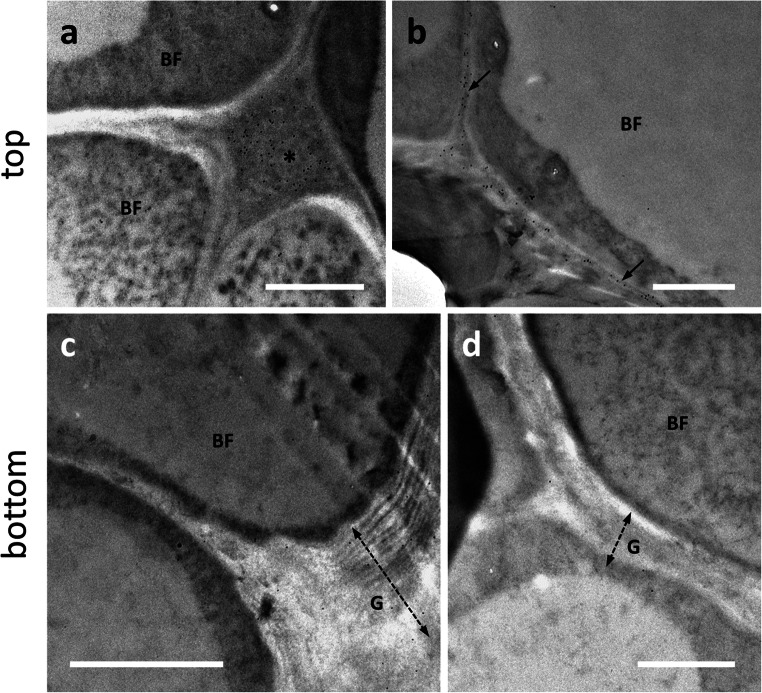
Analysis with JIM7 antibody directed against methyl-esterified pectins. **a**–**b** Top internode of nettle stem. **a** Two developing bast fibers (BF). The JIM7 antibody signal is not detectable, neither in the cytoplasm nor in the cell wall. In contrast, the signal is very clear in the intercellular space (asterisk). Bar: 1000 nm. **b** Another example of a developing bast fiber (BF). The JIM7 signal is observed at the level of the middle lamella that separates the bast fiber from adjacent cells (arrows). Bar 1000 nm. **c**–**d** In the bottom internodes of the nettle stem, the JIM7 signal was rarely detected. Bars: 2000 nm. Double dashed arrows indicate the thickness of the G-layer, which is very large in **c** because of the cutting angle

The analysis with the JIM5 antibody was then complemented by examination with the JIM7 antibody directed against methyl-esterified pectins. In this case, the signal was not readily observable, especially in the bottom internode. In contrast, in the top internode, the JIM7 signal was more readily detectable. Indeed, at the level of developing bast fibers (BF), the signal was present in the intercellular spaces (Fig. 9a, asterisk). In addition, the epitope of JIM7 is likely present in the middle lamella, as suggested by Fig. 9b (arrows) where gold particles were precisely distributed at the border between two adjacent cells. In contrast, in the bottom internode, the signal was hardly detectable (Fig. 9c–d). Only a few gold particles could be observed. Inspection of many sample sections showed almost no evidence of JIM7 signal in the thick G-layer. This result is supported by confocal microscopy observations with LM20 (which recognizes methyl-esterified homogalacturonan): the corners of cells in the pith parenchyma are clearly visible in the bottom internode (Supplementary Fig. S4). The data obtained with JIM7 clearly indicate the absence of methyl-esterified pectins at the level of differentiated bast fibers.

## Discussion

**Fig. 10:**
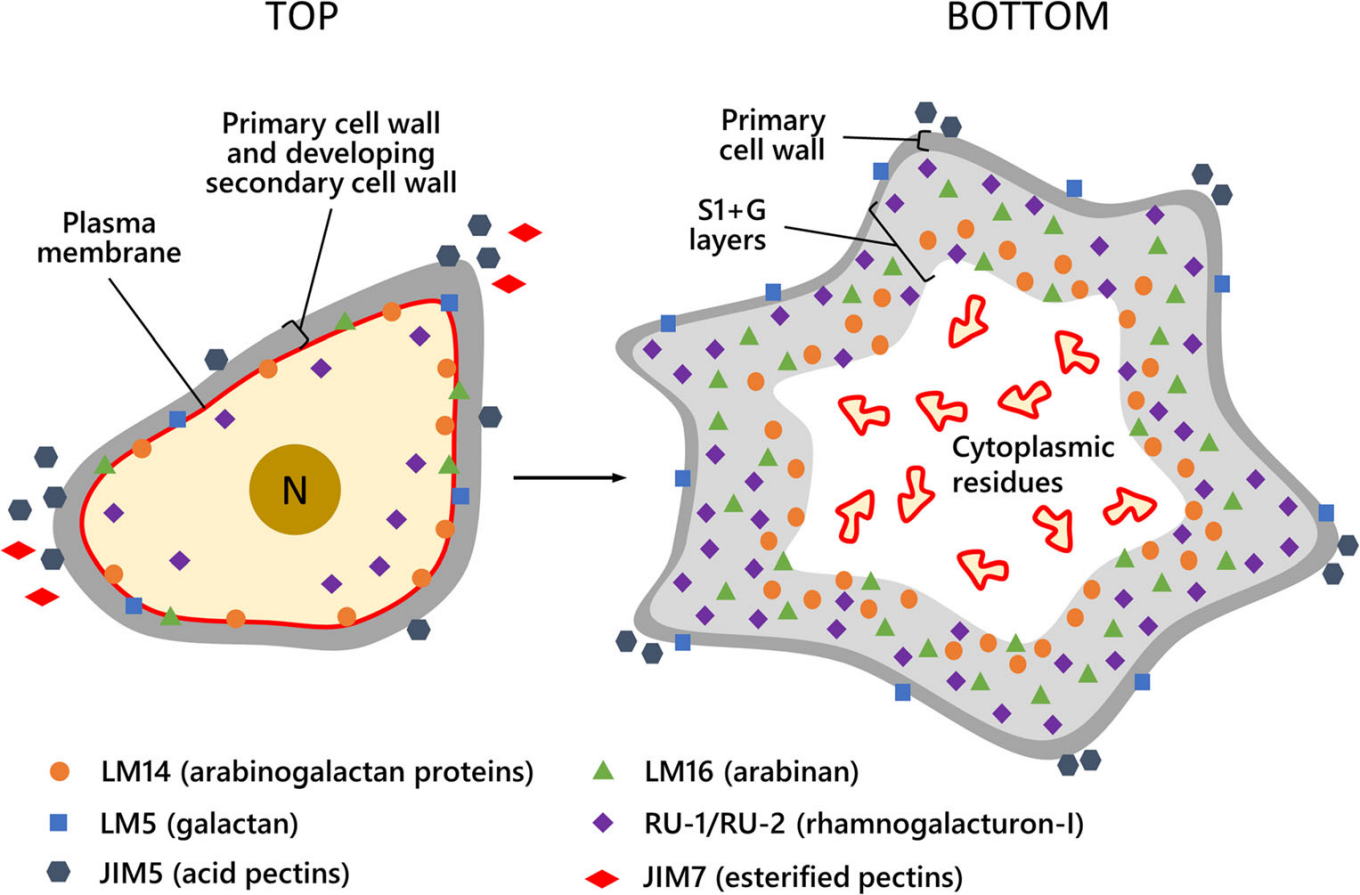
Simplified illustration of the distribution of polysaccharides analyzed in this work. Left, a developing bast fiber as found at the top internode; right, a bast fiber typical of the bottom internode. Polysaccharides and AGPs described in this work are represented by symbols, including the name of the antibody probe used to visualize them. N, nucleus

This study represents, to the best of our knowledge, the first report on the ultrastructural distribution of non-cellulosic cell wall polysaccharides in the stem tissues of a fiber-clone of *U. dioica*, a neglected but potential multi-purpose herbaceous plant. In this manuscript, we provide information on the ultrastructural changes that accompany the development of nettle stems by examining the distribution of chief non-cellulosic polysaccharide classes (galactans, arabinans, pectins), as well as associated proteins (arabinogalactan proteins), in the cell walls of bast fibers. The results obtained are schematically summarized in Fig. 10. To elucidate the development of bast fibers in the nettle stem, we focused on two distinct regions: the one below the shoot meristem (defined as top), characterized by active elongation and primary growth, and an older region (defined as bottom) where the tissues cease to elongate, thicken, and lignify and the bast fibers have developed a thick G-layer. In a manner analogous to what is previously shown in hemp (Guerriero et al. 2017), we decided to focus on internodes characterized by distinctive features in terms of bast fiber development and vascular tissue differentiation.

Bast fibers of nettle are characterized by a flaking multi-layered cell wall; this feature is particularly noticeable in the case of nettle bast fibers even if the term “multilayered” has already been used in other cases such as in the G-layer of hemp phloem fibers (Kiyoto et al. 2018). The multi-layered flaking appearance in nettle suggests a lower compactness of the cell wall. We have already provided preliminary indications on the distribution of cellulose and xylans (Xu et al. 2019) in nettle internodes. The LM10 antibody against unsubstituted and relatively low substituted xylans showed no signal in the bast fibers. The absence of xylans in the S1 layer should be confirmed using other probes, such as LM11 that worked for flax and tobacco bast fibers (McCartney et al. 2005). However, it should be considered that chemical analysis of the cell wall (Xu et al. 2019) suggested that the main hemicellulose of nettle bast fibers sampled from older internodes are xyloglucans, given the high amounts of glucose, fucose, and galactose, in addition to xylose. The reason for the lack of LM10 signal could be due to the non-accessibility of the non-reducing ends of xylans (possibly due to masking caused by interaction with other cell wall components) (Ruprecht et al. 2017; Meents et al. 2019). Therefore, the combined LM10 and LM11 results could provide insight into the chemical nature of the xylans possibly present in nettle bast fibers (substitution, modification of the non-reducing ends, or their masking by interactions). The CBM3a probe showed the presence of crystalline cellulose in the cell wall of xylem cells and in the PCW of parenchyma cells. However, the signal was absent in developing bast fibers. In contrast, more differentiated bast fibers showed a thick cell wall labeled by CBM3a (Xu et al. 2019). Therefore, crystalline cellulose is a typical component of the G-layer of differentiated bast fibers.

In other species, such as flax, non-cellulosic polysaccharides contribute to define the orientation of cellulose fibrils. The secretion of galactans plays a role in the axial orientation of cellulose microfibrils, while their subsequent cross-linking is in relation to the assembly of the G-layer (Gorshkova and Morvan 2006). In flax bast fibers, the main non-cellulosic component of gelatinous cell walls is galactan, and indeed, the G-layer is labeled by the LM5 antibody (Salnikov et al. 2008). In developing fiber cells of flax, the LM5 antibody cross-reacts with epitopes mainly located in the cell wall region closest to the plasma membrane, indicative of a secretion process that accumulates galactans in developing cell wall regions (Andeme-Onzighi et al. 2000; Gorshkova and Morvan 2006; Roach et al. 2011). Still in flax, a galactan-rich cell wall matrix (the so-called Gn-layer) is gradually modified to become the G-layer, rich in crystalline cellulose (Gorshkova and Morvan 2006; Chernova et al. 2007; Salnikov et al. 2008; Roach et al. 2011). In hemp, conversely, galactans recognized by LM5 have not been detected in the G-layer (Blake et al. 2008; Chernova et al. 2018; Behr et al. 2019) and have instead been observed in the cell walls of parenchyma cells. Although the absence of signal can be explained by the “masking” hypothesis, it is also plausible that the content of galactans in the cell wall is extremely low and so undetectable. Like hemp, bast fibers of nettle stems do not show cross-reactivity with LM5 (Fig. 3).

The LM13 and LM14 antibodies are directed against epitopes containing neutral sugars such as arabinose and galactose, which are abundant in the side chains of pectins and hydroxyproline-rich glycoproteins (HRGPs). AGPs are a subfamily of HRGPs, they are ubiquitous in the plant kingdom, from bryophytes to angiosperms. AGPs are commonly composed of a protein backbone and by large, highly branched glycan chains rich in arabinose and galactose. It is one of the most heterogeneous and complex families of macromolecules, capable of performing different and multiple functions. AGPs are found at the interface between plasma membrane and cell wall and take part in processes such as plant growth, development, and reproduction. In addition, AGPs can also act in response to biotic and abiotic stress (Ellis et al. 2010; Le Gall et al. 2015; Mareri et al. 2019). AGPs have been also proposed to function as “biochemical turgor sensors” capable of sensing the environment and relaying information to other plasma membrane proteins. Thus, they could help regulate the deposition of various cell wall polysaccharides thereby affecting cell wall structure and mechanical properties. As an example, we can cite the pollen tube, a not only tip-growing but also intrusively growing cell in that it grows by making its way between pistil cells. In this case, perturbation of AGPs changes pectin localization and impacts cell wall stiffness (Leszczuk et al. 2019). In higher plants, AGPs also include proteins belonging to the family of fasciclin-like arabinogalactan (FLA) proteins. Expression of *FLA*s correlates with secondary wall cellulose synthesis in *Arabidopsis* stems, and with wood formation in tree stems and branches. In textile hemp, specific groups of *FLA*s were over-expressed in bast fibers and their promoters were found to contain motifs putatively recognized by MYB3, a repressor of phenylpropanoid biosynthetic genes (Guerriero et al. 2017). *FLA*-knockout plants of *Eucalyptus* and *Arabidopsis* showed alterations in stem biomechanics as well as cell wall architecture and composition with reduced arabinose, galactose, and cellulose levels (MacMillan et al. 2010). This suggests that FLAs contribute to plant stem strength by impacting cellulose deposition. The antibody LM14 binds to some extent to all cell walls in stem sections of *Arabidopsis thaliana*, but not in tobacco stems (Moller et al. 2008). In addition, the antigen detected by LM14 is reported to increase in cold-stressed banana stems and roots (Yan et al. 2015). In hemp, immunohistochemical analysis carried out with LM14 revealed that the epitope is distributed ubiquitously in different tissues of the hemp stem, suggesting a wide distribution of these AGPs in stem tissues (Guerriero et al. 2017). In this manuscript, we used two methodological approaches to analyze the distribution of LM14 epitope: immune electron and confocal microscopy. They did not provide fully matching results, as the latter revealed a widespread distribution of LM14 epitopes (see Supplementary Fig. S2); however, the two techniques are different in sensitivity, a factor that may explain the differences detected between the two microscopy approaches. Both techniques, however, highlighted a more intense labeling on the inner side of the cell wall (Fig. 5 and Supplementary Fig. S1). The relative abundance of the epitope in the cell wall region closest to the plasma membrane suggests that it is secreted during the progressive development and thickening of the G-layer. Therefore, a role for the LM14-recognized antigen as a biomechanical stress sensor cannot be ruled out.

The neutral side chains associated with rhamnogalacturonan-I (RG-I) can also be extraordinarily complex. Arabinans and arabino-galactans are polymers responsible for the formation of “pores” in cell walls due to their ability to not only interact with water but can also associate with cellulose (Verhertbruggen et al. 2009). In this way, arabinans can contribute to the properties of the cell wall in terms of matrix porosity and mechanical properties. In the latter case, arabinans can be important for supporting the flexibility of cell walls; an emblematic example is represented by resurrection plants (Moore et al. 2013).

The various forms of arabinans can be studied using antibodies that discriminate against various arabinan-based structures; among them, the antibody LM16 binds to branched arabinan (Verhertbruggen et al. 2009). In *Miscanthus* stem, the LM16 antibody labeled similarly all regions except the phloem. Branched arabinans were detected in the parenchymatic cell walls and in the inner region of the SCWs (Cao et al. 2014). In parenchyma cells of the *Arabidopsis* stem, the LM16 antibody labeled the cell wall areas facing each other (adhesion areas). In contrast, in tobacco stem sections, the signal was observed only in xylem and phloem cells (Pauly and Keegstra 2010). The analysis of arabinans in nettle stems, carried out using the LM16 antibody, highlighted an intense accumulation of arabinofuranosidase-processed arabinans in the thick G-layer of bast fibers. The signal was spread throughout the entire cell wall thickness and in the cytoplasm as aggregates around endomembrane residues (Fig. 7a and b). After cell differentiation, the LM16 signal accumulated on the cytoplasmic side of the cell wall. In contrast, in young region at the top of nettle stem, the LM16 signal was much less intense and observable on the inner side of developing bast fibers (Fig. 8). The comparison between the two developmental stages suggests that the accumulation of LM16-labeled arabinans is necessary for the correct structure of bast fibers’ walls. However, the preferential occurrence on the side of the cell wall near the plasma membrane indicates an accumulation resulting from secretion of branched arabinans.

Pectins are structurally and functionally the most complex polysaccharides of plant cell walls. They have functions during growth, in the establishment of morphology, in development, and defense of plants; they also function as a gelling and stabilizing polymer in various food products (Voragen et al. 2009; Palin and Geitmann 2012; Saffer 2018). Pectins are a family of polysaccharides rich in galacturonic acid, including homogalacturonan, rhamnogalacturonan I, the substituted galacturonans rhamnogalacturonan II (RG-II), and xylogalacturonan (Willats et al. 2001; Caffall and Mohnen 2009). Pectins present in the G-layer have been studied using two monoclonal antibodies (INRA-RU1 and INRA-RU2) (Ralet et al. 2010) capable of cross-reacting with the backbone of rhamnogalacturonan I, without showing cross-reactivity to homogalacturonans, galactans, or arabinans. The RU1 antibody recognizes the R7-U7 pattern (seven rhamnose-galacturonic acid repetitions) and shows reactivity towards R6U6, R8U8, and R9U9 (Ralet et al. 2010). In the bottom region of the nettle stem, RU1 clearly labels fully developed bast fibers, which show an intense, evenly distributed signal throughout the cell wall (Fig. 9). In contrast, the RU1 signal in the top sections of nettle stem distributed differently than in bottom regions. In cells corresponding to bast fibers at an early developmental stage, we observed a cytoplasmic signal. Therefore, we believe that the RU1 signal is prevalent in the cytoplasm and the inner side of the cell wall in the top region, while in the bottom internode, the signal is prevalent throughout the whole bast fibers’ wall. Thus, bast fiber development requires a progressive deposition of RG-I starting from an initial clustering in the cytoplasm (which probably anticipates secretion) followed by an accumulation in the cell wall inner side and then a progressive diffusion through the entire cell wall. Labeling of bast fibers with thicker G-layers by RU1 is stronger than RU2. This observation agrees with the results on flax hypocotyls (Ralet et al. 2010). The two antibodies have a different specificity regarding the number of repeating disaccharides, therefore the difference in signal intensity could indicate the distribution of the neutral side chains on the RG-I backbone. In flax fibers, the main non-cellulosic component of gelatinous cell walls is galactan, which is part of RG-I strongly associated with cellulose. Still in flax, the development of the gelatinous cell wall is coupled with the accumulation of Golgi vesicles fusing with the plasma membrane (Chernova et al. 2018). A similar process can also be hypothesized in nettle considering the predominant cytoplasmic distribution of RU1 antigens in the top regions. For further comparison, in hemp at H20 stage, the G-layer is clearly labeled by the RU1 antibody while the “stripped” layer in H20 shows cross-linking with the INRA-RU2 antibody (Behr et al. 2019). While RG-I in the middle lamella functions as a kind of adhesive between neighboring cells, in the G-layer, it likely contributes to a higher degree of wall compaction through lateral interaction with cellulose microfibrils (Mikshina et al. 2015). Chemical analysis of the nettle stem cell wall already showed a difference in the structure and composition of rhamnogalacturonan in bast fibers, highlighted by prominent levels of galactose and the presence of the Ca^2+^-pectate gels (Xu et al. 2019).

The hypothesis that pectins function as a glue between adjacent cells is also reinforced by the experimental evidence obtained with the JIM5 antibody directed against acidic pectins. The signal obtained by JIM5 localized in intercellular spaces or in the middle lamella, which implies a role in the adhesion or contact between adjacent cells. The hypothesis that pectins are an integral part of the adhesive system joining adjacent cells has been reviewed (Zamil and Geitmann 2017). A comparison of the top and bottom sections of nettle stem showed no difference. In both cases, the JIM5 signal is confined to intercellular spaces and, occasionally, to the middle lamella (Fig. 8). This shows no modification of the distribution pattern of the JIM5 antigen and therefore suggests that, in bast fibers, the distribution of acidic pectins does not change during secondary growth in nettle stems. Similarly, we found no significant differences in the distribution of esterified pectins as labeled by JIM7. We interpret this finding as further confirmation that homogalacturonans (acidic or esterified) do not play a specific role in the maturation of bast fibers. Although we have not measured the signal density of JIM5, it seems quite clear that there are no significant differences, at least in the intercellular zones, between bast fibers and surrounding cells. Although this requires further experimental validation, the immune electron microscopy results indicate that most of the methyl-esterified pectins are quickly converted into the cell wall, thus generating acidic pectins, both at the top and bottom levels. This type of pectin distribution distinguishes this result from other data described in the literature. For example, changes in distribution, quantity, and type of pectins are responsible for several developmental processes, such as the ripening of fruits. In such cases, it is possible to correlate pectin changes with the development of a given tissue or organ. In the specific case of fruit, the disassembly of the PCWs and middle lamella takes place through an altered expression or activity of the enzyme pectin-methyl esterase and polygalacturonase, resulting in a softening of the pericarp (Hyodo et al. 2013). Pectin-methyl esterase catalyzes the de-esterification of pectins and the analysis of its distribution has often provided information on pectin modification sites, especially in the transition between methyl-esterified and acidic pectins (Morvan et al. 1998).

Ultimately, the data in this manuscript and those in Xu et al. (2019) indicate that the differences in nettle bast fibers between young and older stem internodes relate to an increase in crystalline cellulose deposition. Xylans detected by LM10 are associated with the xylem tissue and not with the outer layer of the bast fiber’s G-layer, differently from hemp (Behr et al. 2019); galactans detected by LM5 also do not appear to contribute to the differentiation process of G-layer. In contrast, arabinans labeled by LM16 appear to accumulate concomitantly with the progressive thickening of the G-layer, as do pectins of the rhamnogalacturonan I type. In contrast, homogalacturonan-type pectins (both esterified and acidic) are almost absent and restricted to intercellular spaces or the middle lamella. Similarly, AGPs recognized by LM14 accumulate as thickening of the G-layer progresses, although they are more abundant on the cytoplasmic side of the cell wall.

## Supplementary Information

ESM 1 (PDF 760 kb)

## Data Availability

Not applicable.
