# Supplementary material for: Immunohistochemical analyses on two distinct internodes of stinging nettle show different distribution of polysaccharides and proteins in the cell walls of bast fibers
Source: Protoplasma. 2021 Apr 10;259(1):75–90. doi: 10.1007/s00709-021-01641-1 (PMC8752570; doi:10.1007/s00709-021-01641-1)
Supplement: Supplementary file 1 — (PDF 760 kb) [file 709_2021_1641_MOESM1_ESM.pdf]

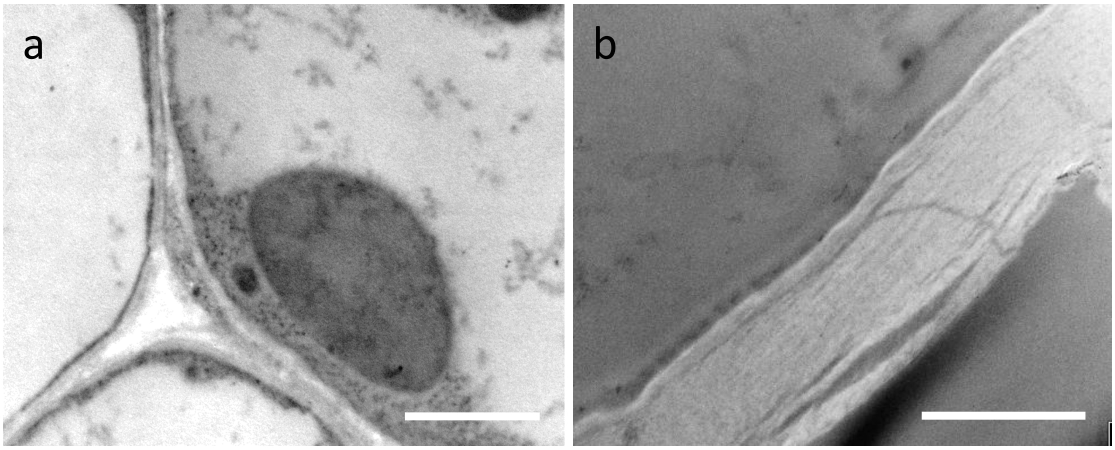

Supplementary Figure S1: Controls of immunogold labelling as carried out by omitting the primary antibodies; no signals were detected due to the secondary antibodies. (a) Detail of a developing bast fiber at the top internode. (b) Detail of the G-layer of a bast fiber sampled at the bottom internode. Bars 2 µm

Immunohistochemical analyses on two distinct internodes of stinging nettle show different distribution of polysaccharides and proteins in the cell walls of bast fibers

Protoplasma

Claudia Faleri, Xuan Xu, Lavinia Mareri, Jean-Francois Hausman, Giampiero Cai *, Gea Guerriero

* Corresponding author: Dipartimento Scienze della Vita, University of Siena, via Mattioli 4, Siena, Italy; cai@unisi.it

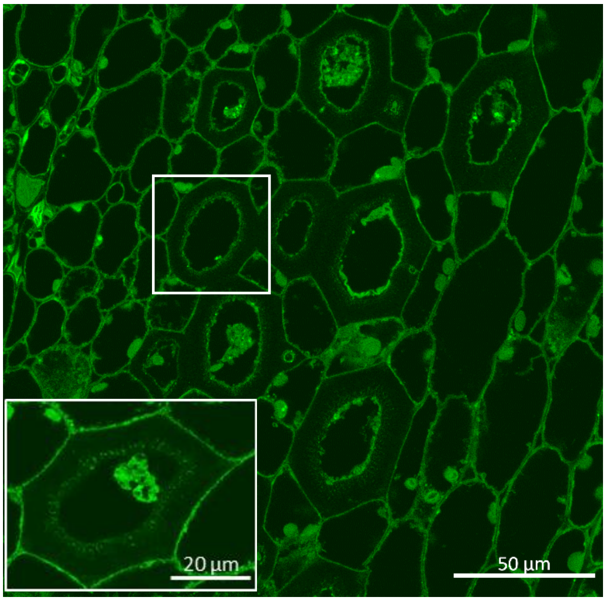

Supplementary Figure S2: Immunodetection of the LM14 epitope specific for AGPs in the bottom internode of the nettle stem. The white square indicates one bast fiber. Inset: detail of a magnified bast fiber. The length of scalebar is indicated in the figure.

Immunohistochemical analyses on two distinct internodes of stinging nettle show different distribution of polysaccharides and proteins in the cell walls of bast fibers

Protoplasma

Claudia Faleri, Xuan Xu, Lavinia Mareri, Jean-Francois Hausman, Giampiero Cai *, Gea Guerriero

* Corresponding author: Dipartimento Scienze della Vita, University of Siena, via Mattioli 4, Siena, Italy; cai@unisi.it

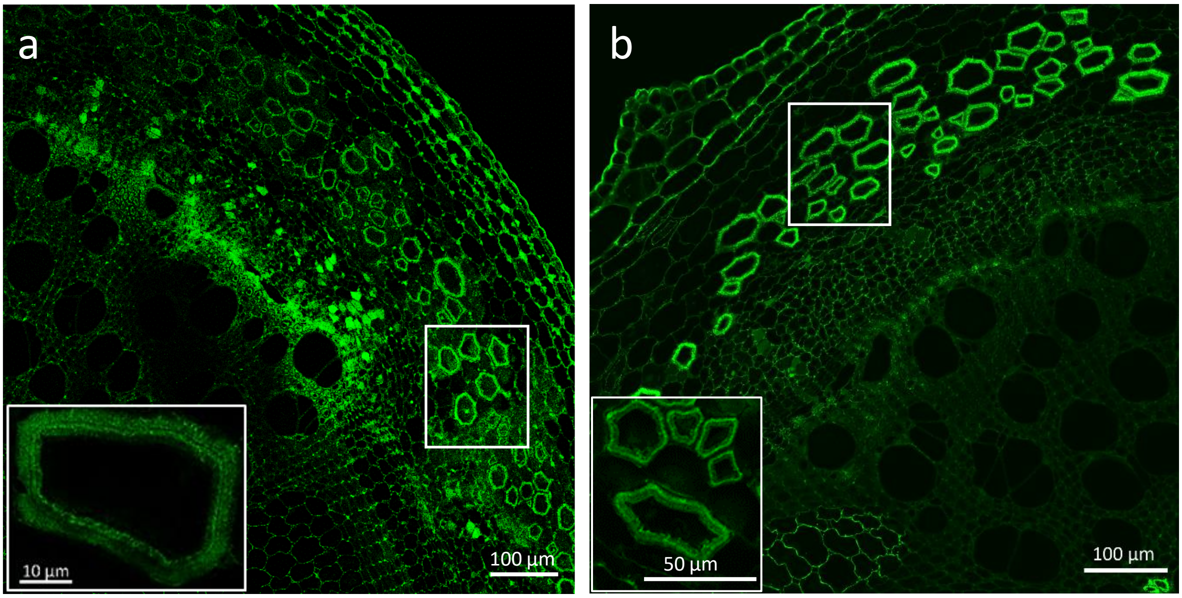

Supplementary Figure S3: Immunodetection of the RU1 (a) and RU2 epitope (b) specific for the RGI backbone in the bottom internode of the nettle stem. The white squares indicate one bast fiber. Inset: detail of a magnified bast fiber. The length of scalebar is indicated in the figure.

Immunohistochemical analyses on two distinct internodes of stinging nettle show different distribution of polysaccharides and proteins in the cell walls of bast fibers

Protoplasma

Claudia Faleri, Xuan Xu, Lavinia Mareri, Jean-Francois Hausman, Giampiero Cai *, Gea Guerriero

* Corresponding author: Dipartimento Scienze della Vita, University of Siena, via Mattioli 4, Siena, Italy; cai@unisi.it

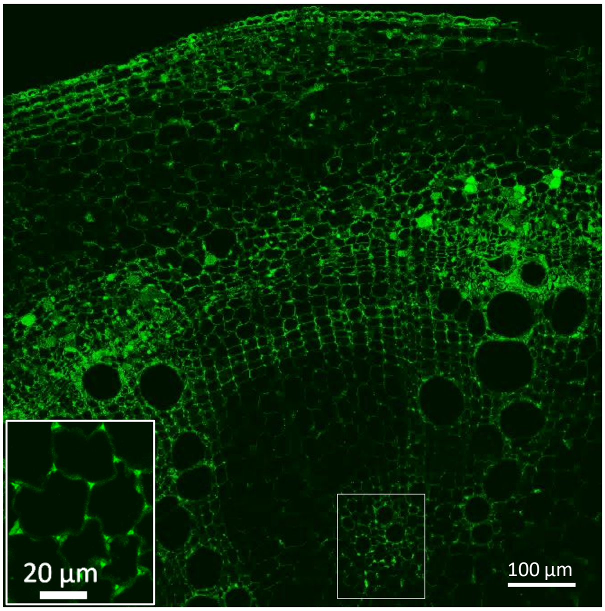

Supplementary Figure S4: Immunodetection of the LM20 epitope specific for methyl-esterified homogalacturonan in the bottom internode of the nettle stem. The white square indicates the pith parenchyma. Inset: detail of a magnified parenchymatic cell in the central pith. The length of scalebar is indicated in the figure.

Immunohistochemical analyses on two distinct internodes of stinging nettle show different distribution of polysaccharides and proteins in the cell walls of bast fibers

Protoplasma

Claudia Faleri, Xuan Xu, Lavinia Mareri, Jean-Francois Hausman, Giampiero Cai *, Gea Guerriero

* Corresponding author: Dipartimento Scienze della Vita, University of Siena, via Mattioli 4, Siena, Italy; cai@unisi.it
